# Insight into the effect of low temperature treatment on trichome density and related differentially expressed genes in Chinese cabbage

**DOI:** 10.1371/journal.pone.0274530

**Published:** 2022-09-15

**Authors:** Peixia Sun, Chuang Huang, Liping Zhang, Dan Wu, Wei Zhang, Shuang Yu, Genying Fu, Shanhan Cheng, Zhiwei Wang, Qin Deng, Guopeng Zhu, Pingwu Liu

**Affiliations:** 1 Fang Zhiyuan Academician Team Innovation Center of Hainan Province, Key Laboratory for Quality Regulation of Tropical Horticultural Crops of Hainan Province, School of Horticulture, Hainan University, Haikou, China; 2 Hainan Yazhou Bay Seed Laboratory, Sanya Nanfan Research Institute of Hainan University, Sanya, China; Huazhong University of Science and Technology, CHINA

## Abstract

Trichome is important for help plant resist adversity and external damage. However, it often affects the appearance and taste of vegetables. In the present study, the trichome density of leaves from two Chinese cabbage cultivars with and without trichomes treated at low temperature are analyzed by biological microscope, and the differentially expressed genes related to trichomes formation were screened through transcriptome sequencing. The results showed that the number of leaves trichomes was reduced by 34.7% at low temperature compared with room temperature. A total of 661 differentially expression genes effecting trichomes formation were identified at the CT vs C, LCT vs LC, CT vs LCT. Several differentially expression genes from every comparison group were enriched in plant hormone signal transduction and amino acid biosynthesis pathway. Combined with the central genes obtained by WGCNA analysis, five candidate genes *Bra029778*, *Bra026393*, *Bra030270*, *Bra037264* and *Bra009655* were screened. qRT-PCR analysis verified that the gene expression differences were in line with the trend of transcriptome data. This study not only found possible new key genes and laid a foundation for revealing the molecular mechanism regulating the formation of trichome in Chinese cabbage, but also provided a new way to study plant surface trichomes.

## Introduction

Trichomes are special structures on the surfaces of plants derived from epidermal cells [1, 2], and its density and size are determined by the stage and location of growth and development in plants [3]. Trichomes not only can secrete some lipids or secondary metabolites, such as changing the body and feces odor of feeding insect larvae to attract ants to prey on them [4, 5], but secrete some volatile organic compounds to attract parasitic wasps and other insect natural enemies to prey on pests. Furthermore, trichomes play a crucial role in defending against pests or bacteria [6]. It is often an important factor in response to drought, water, salt, high temperature and other abiotic stresses [7–9]. For example, the total glandular hair density on both sides of leaves of *schizonepeta tenuifoliabriq* chinensis was significantly increased in response to salt stress [10], and the reduction of steam pressure difference on leaf surface resulted in the decrease of glandular hair density on leaves of Birch [11]. In addition, the trichome secretions are used as raw materials for spices, medicines, food additives, resins and essential oils [12, 13], which have important ecological and economic value to human beings.

Chinese cabbage is an important vegetable in Cruciferae, rich in vitamins, crude fiber, carotene and other nutrients. It is the vegetable with the largest cultivation area, the most daily consumption and the most popular among consumers in China. Chinese cabbage can be divided into trichomes and without trichomes types. Although Chinese cabbage trichomes play an obvious role in increasing leaf thickness to reduce heat of epidermis, loss of water, and defense against insect pathogen invasion and mechanical injury [14, 15], it also affects the consumption quality, and sometimes even cause stabbing injury to human body. Therefore, how to remove trichomes or regulate the formation of trichomes is extremely important for fruit and vegetables breeding. It was found that simulated summer heating increased the number of glandular hairs of *Empetrum nigrum* [16]. The decrease oftemperature reduced the density of glandular hairs in the leaves of *Origanum vulgare* [17]. Hormone treatment showed that GA_3_ and MeJA could significantly increase the number and density of tomato (*Solanum lycopersicum*) and *Arabidopsis* trichome [18, 19], and ethylene treatment increased epidermal branching in cucumber [20], while SA treatment inhibited epidermal branching in *Arabidopsis thaliana*. It was found that jasmonate promoted the development of *Arabidopsis thaliana* epidermis by inhibiting the interaction between jasmonate ZIM protein and GL1 and EGL3/GL3 in the transcriptional regulatory complex [21–23]. The transcription factor *MYB23* gene was involved in controlling trichome branching and leaf edge trichome initiation [24–26]. Moreover, gibberellin, cytokinin and ethylene controlled the formation of epidermal and root hair cells in *Arabidopsis thaliana* through GIS family and subfamily genes [27].

Li located *BraGL1* gene controlling leaf trichome in Chinese cabbage and found that the DNA- binding domain of *Brassica GL1* was highly homologous with *Arabidopsis thaliana* [28, 29]. A gene located on chromosome 6 of Chinese cabbage was found functionally complementary to the *ttgll* mutant of *Arabidopsis thaliana* and affected the formation of trichomes [30]. The major gene located on A09 through CAPS markers and RAD-seq, contributed 78% to the number of trichomes in Chinese cabbage leaves [31]. The *BraGL1* gene in Chinese cabbage and showed that *BraGL1* was the cause of the absence of trichomes in Chinese cabbage. Furthermore, 266 genes including *GL3*, *EGL1*, *SAD2* genes and *WRKY*, *MYB*, *NAC* transcription factors that might be related to the development of leaf trichomes were screened from the differential expression profiles of F_2_ generation trichome leaves without trichomes and with trichomes [32]. The number of candidate genes that affect the density of Chinese cabbage trichomes is still limited, especially the mechanism of low temperature inhibiting the formation of Chinese cabbage trichomes remains elusive.

The purpose of this study is to investigate the phenotypic changes and molecular mechanisms of leaf trichomes in Chinese cabbage in response to low temperature, and to explore a new pathway for the regulation of plant trichomes.

## Materials and methods

### Materials

The materials used for phenotypic and transcriptomic determination were Chuntai Chinese cabbage (CT, trichome) (purchased from Guangdong Superior Seed Import Service Company) and Chaozhou Kuai Da Xia Huang Bai (C, without trichome) (purchased from Shantou Jinxuan Seed Industry Company). CT and C are treated at room temperature, while Low temperature treatment with trichomes is denoted as LCT, low temperature treatment without trichomes is denoted as LC.

### Plant culture

The full seeds were selected and placed in a petri dish covered with two layers of filter paper. The filter paper was soaked with distilled water and germinated in a light incubator at 22±1°C. After germinating and whitening, the seeds are moved to a 7×7 cm pot and placed in a light incubator simulated room temperature (temperature: 27/21°C) when growing to 4 true leaves, 20 seedlings with the same size and growth trend were selected and transferred to a low temperature (15/9°C) incubator in advance for low temperature treatment. Same number of seedlings were treated at room temperature. All plants are placed under the condition of 16 h/8 h light cycle and 6000 Lx light intensity.

### Sampling and data analysis

After 10 days of treatment at low and room temperature, 3 Chinese cabbage plants were taken from each treatment and 3 leaves were selected from top to bottom. 3 holes were drilled in each leaf with a 9 mm hole punch and 3 fields were selected for each hole. The data were observed and recorded under a 10×10 biological microscope. Excel 2016, GraphPad Prism 5, Adobe Photoshop were used for data collation and mapping, and IBM SPSS Statistics 20 was used for significance test (Duncan, P < 0.05). At the same time, 0.5 g of the expanded third leaf of each plant was collected for three biological repeats for transcriptome detection.

### RNA extraction and library construction

Total RNA was extracted from Chinese cabbage leaves, and the purity and integrity of sample RNA were detected. Now, all Raw date obtained by sequencing have been submitted to NCBI, SRA: PRJNA842790. All clean data were calculated using Q20 and Q30, and clean reads were sequentially compared with the reference genome using HISAT2 V2.0.5 to obtain location information on the reference genome and sequence characteristic information of the sequenced samples. Only perfectly matched reads are used for further analysis and annotation.

### Differentially expressed genes (DEGs)

Differential genes were identified using FPKM (Fragments per kilobase per million reads) and DESeq2 software (1.20.0) was used for differential expression analysis between the two comparison combinations. Adjust the P value (padj) | log^2^FoldChange | threshold for significantly differentially expressed. In order to comprehensively obtain the functional information of differential genes, gene function annotations were carried out through the database, including GO and KEGG. GO and KEGG enrichment analysis of differentially expressed genes were realized by software.

### Construction and visualization of gene network

The R package WGCNA was used to calculate the functional set of weighted association analysis, network construction, gene screening. The co-expression network was visualized using Cytoscape (v.3.7.1) software.

### qRT-PCR was used to verify the differential genes

The extracted RNA was reverse transcribed using HiScript Ⅲ All-in-one RT Super Mix Perfect for qPCR kit. qRT-PCR was performed using ChamQ Universal SYBR qPCR Master Mix kit and Quant Studio 3 Real-time Quantitative PCR System (ABI, USA) instrument. The relative expression level of genes was calculated by 2^-ΔΔCt^ method, and Actin was selected as an internal reference gene. Differential genes were selected and gene-specific primers are shown in Table 1, were designed using Primer V5.0, which were synthesized by Shenggong Bioengineering (Shanghai).

**Table 1:** qRT-PCR primer.

Gene ID	Forward (5’ to 3’)	Reverse (5’ to 3’)
*Bra023654*	GCAACAAGCAAAAGAGCAACC	GCTTGAAATGAAGGTTGGCTC
*Bra039762*	TCAAGTCATACCCGACCAAGAT	CGACTTTCCTCATCGCACC
*Bra003253*	GTGGACAAACTACCGTTGGAAT	TCCTCAACTTACGCTTACGAACT
*Bra029311*	GGCGAGTTTAGTTCAGAGGAGG	TGCGTGGTGGTGACAGTAGG
*Bra035148*	TCGGAGATGCTAACTTTGATGTG	CAATAACCGCTGAACTAACTGCT
*Bra018529*	GCGACGGGTCCTTGATTGT	GAACTTGGCGAAAGAAACAGC
*Bra000809*	CGGTTCCTTTCTCCGTTCA	CCAAAGGCAGCAAAAGTGAA
*Bra025589*	TCCTCGGCAACAGATGGTC	ATCGTCGTCCGTAGTGTCAAA
*Bra023486*	AGGTGGTCCTTGATTGCTAAAA	CAATATCACCATTACTCGGCTTG
*Actin*	ATCTACGAGGGTTATGCT	CCACTGAGGACGATGTTT

## Results

### Influence of low temperature treatment on trichome density of Chinese cabbage

**Fig 1:**
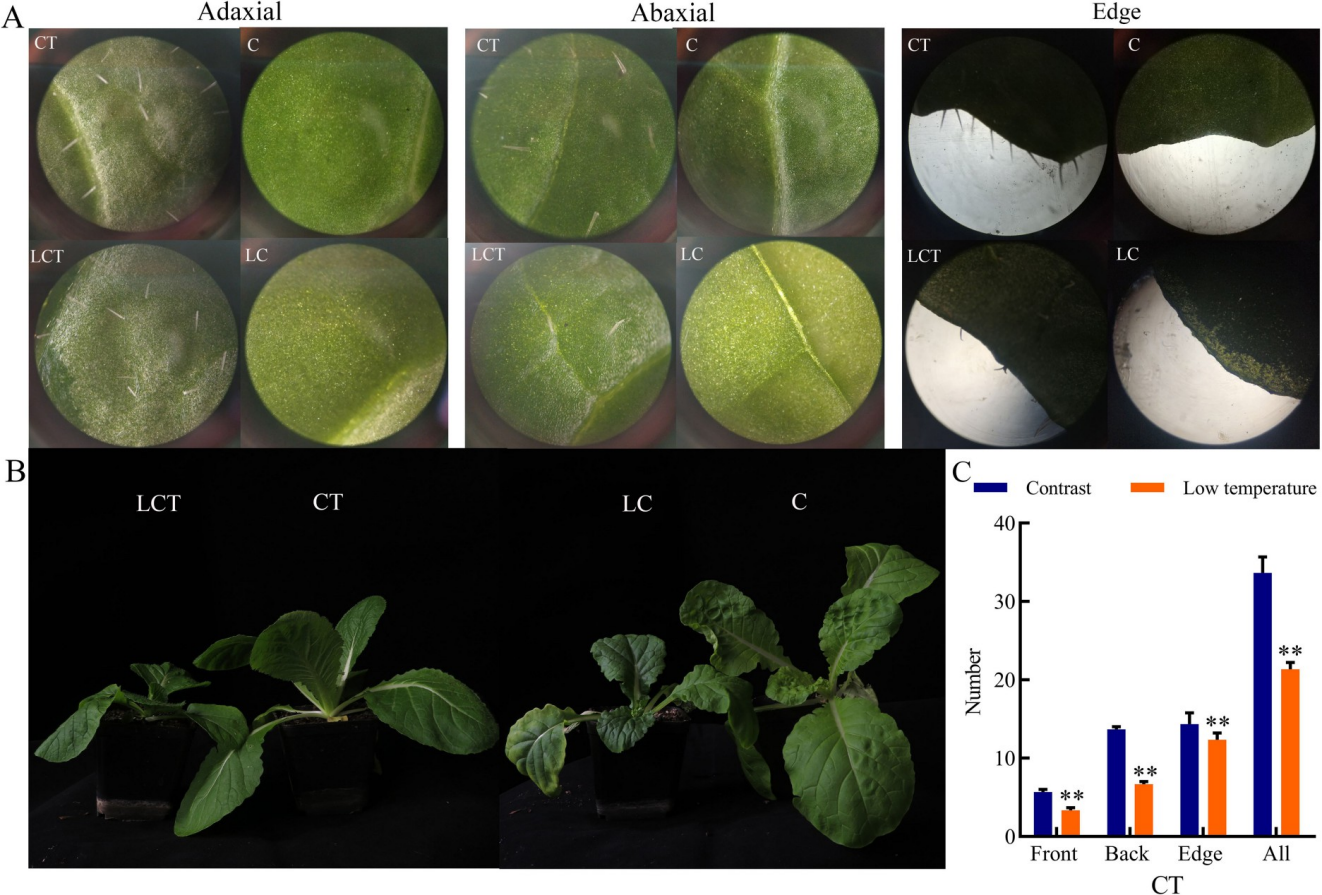
Effect of low temperature treatment on trichomes. A, Three visual fields (adaxial, abaxial, and edge) at room temperature (CT, C) and low temperature (LCT, LC); B, Plants at room temperature (CT, C) and low temperature (LCT, LC); C, Density of trichomes at room temperature (CT) and low temperature (LCT).

The density of trichomes on the adaxial side, abaxial side and edge of leaves of the Chinese cabbage varieties after low-temperature treatment were significantly reduced, among which, the number of trichomes on the abaxial side of leaves was significantly reduced by 53.6%, and the total number of trichomes was 65.3% of room temperature treatment on the three fields (Fig 1A and 1C). The hairless variety C was still hairless before and after low temperature treatment (Fig 1A and 1B).

### Quality control of sequencing data

Refer to Brapa_sequence_V1.0. fasta database in Ensemble Plants to generate raw data in FASTQ format for this sequencing. The mismatch rate of experimental materials is low. The data volume of bases is between 6.1 and 7.3 G, and the proportion of Q20 is above 96%. The base recognition accuracy of Q30 is above 91%, and the GC content is above 45% (Table 2).

**Table 2:** Statistical analysis of RNA-seq reading segments.

Sample	Raw reads	Clean reads	Mapped rate (%)	GC-count (%)	Q20 (%)	Q30 (%)
C1	50397258	48687520	88.6	46.62	98.13	94.64
C2	44962240	43493470	89.07	47.1	98.07	94.56
C3	46282198	44951976	86.1	46.85	98.2	94.77
CT1	44359098	41517946	88.12	45.65	97.85	94.11
CT2	47881796	47568590	87.52	47.86	96.83	91.8
CT3	46095274	44365122	88.51	45.96	98.07	94.51
LC1	42294282	40674090	90.68	47.27	98.24	94.78
LC2	44381250	42900888	90.56	47.61	98.2	94.67
LC3	47910268	47367088	88.4	47.27	97	91.91
LCT1	48549544	48119596	89.64	47.79	96.76	91.62
LCT2	43172328	41278792	89.94	46.43	98.14	94.78
LCT3	43261326	41226126	91.36	46.48	98.27	94.99

**Fig 2:**
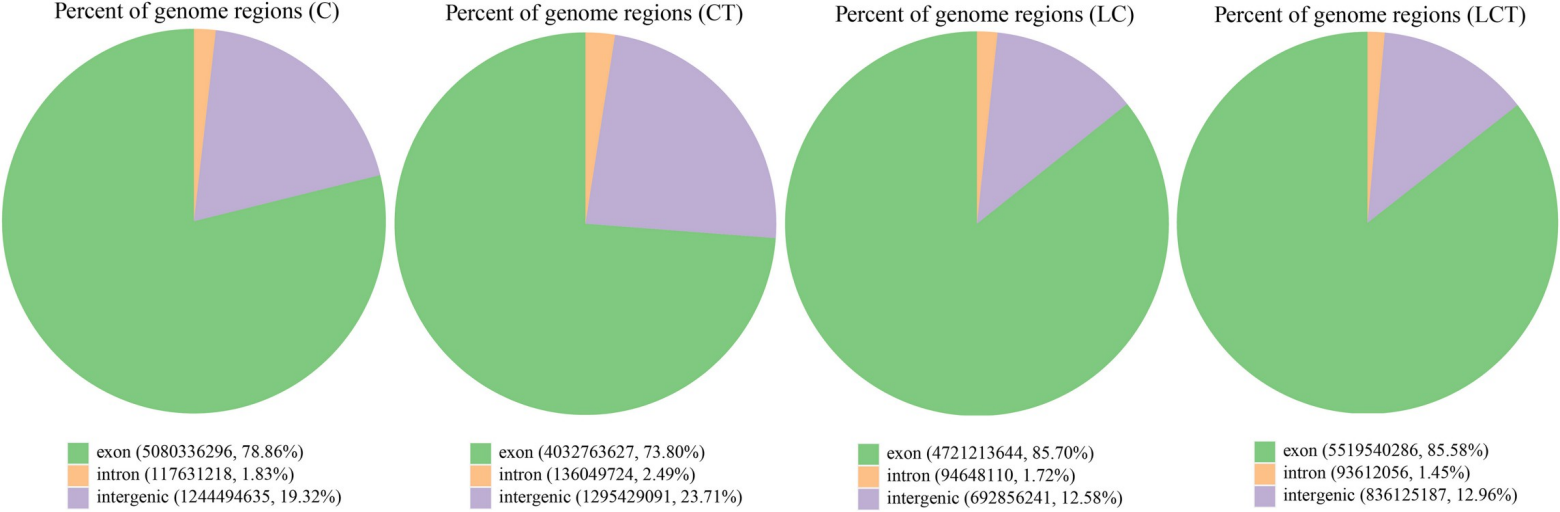
Distribution of reads in different regions of the reference genome.

The position of all reads aligned to the reference genome was statistically analyzed. In C, 78.86% of reads were aligned to exons, 1.83% to introns, and 19.32% to gene spacer sequences. In CT, 73.8% of reads were aligned to exons, 2.49% to introns, and 23.71% to gene intervals. In LC, 85.7% of reads were aligned to exons, 1.72% to introns, and 12.58% to gene intervals. In LCT, 85.58% of reads were aligned to exons, 1.45% to introns, and 12.96% to gene intervals (Fig 2).

### Analysis of related expression levels

**Fig 3:**
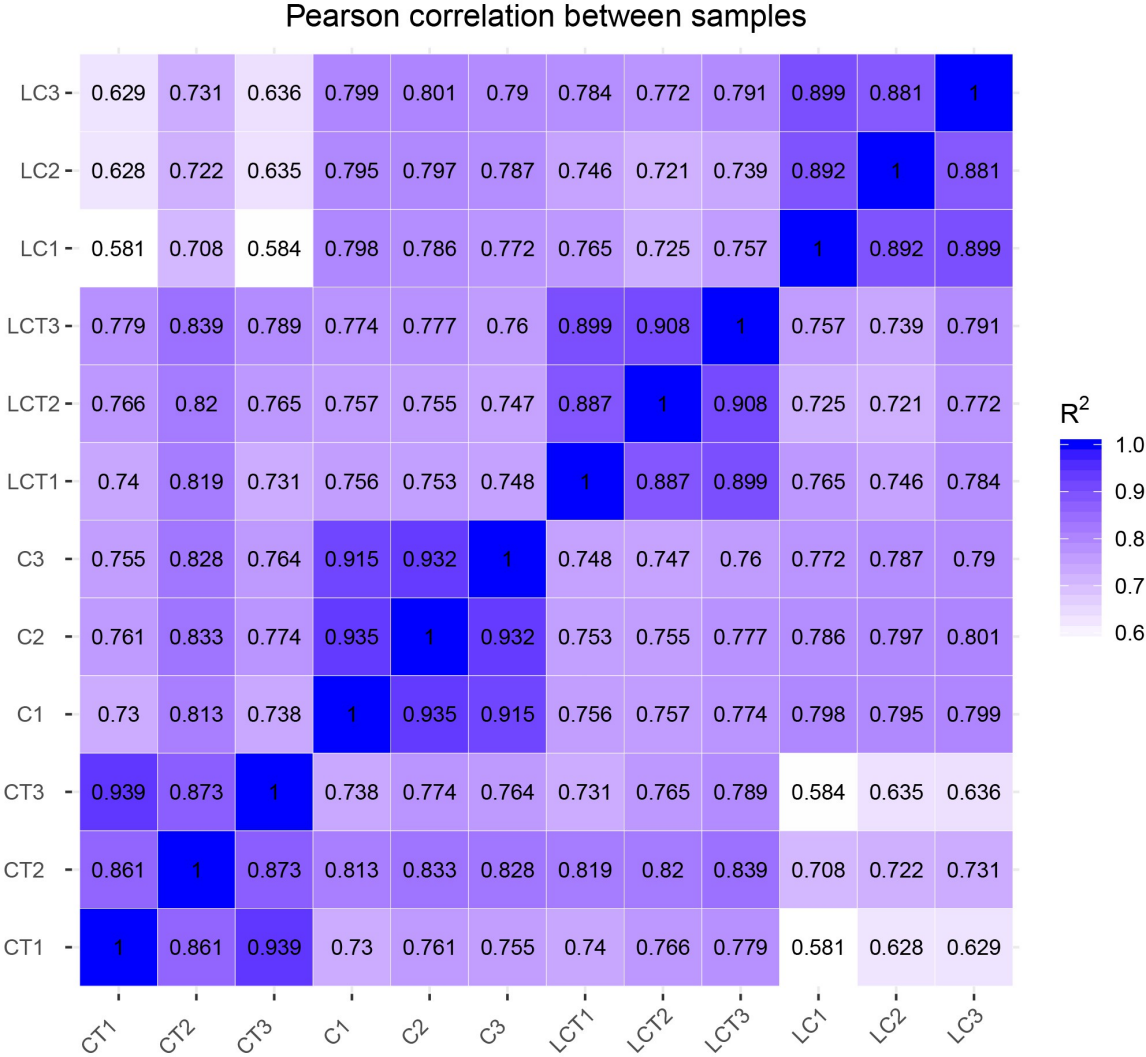
Correlation heat map between samples.

DESeq2 software was used to analyze the expression levels of each sample of Chinese cabbage treated at low temperature. It has been seen from the correlation heat map of each treatment and repetition that the sample has good correlation, which can be further analyzed (Fig 3).

### Analysis of differentially expressed genes

**Fig 4:**
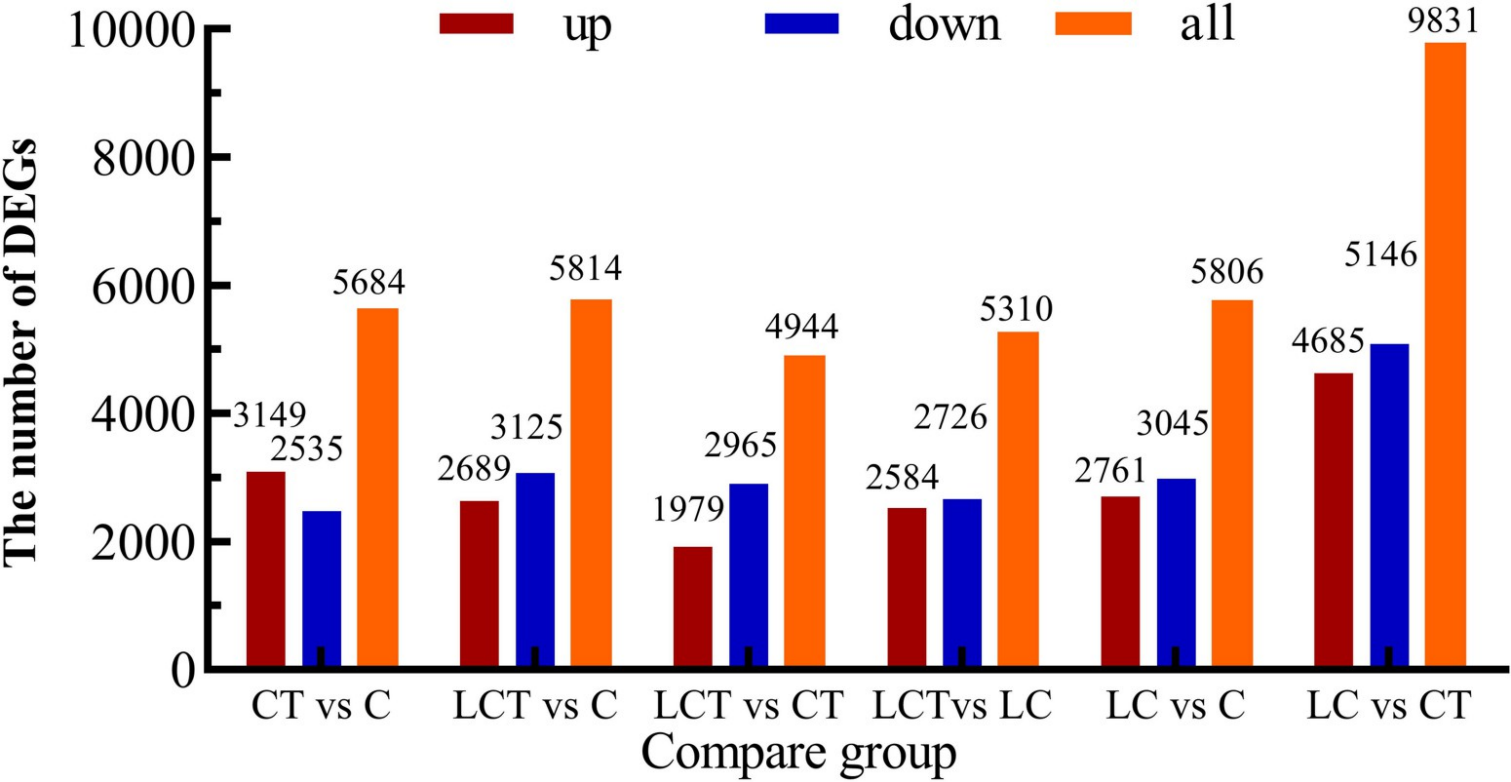
Statistical histogram of the number of genes in different combinations.

**Fig 5:**
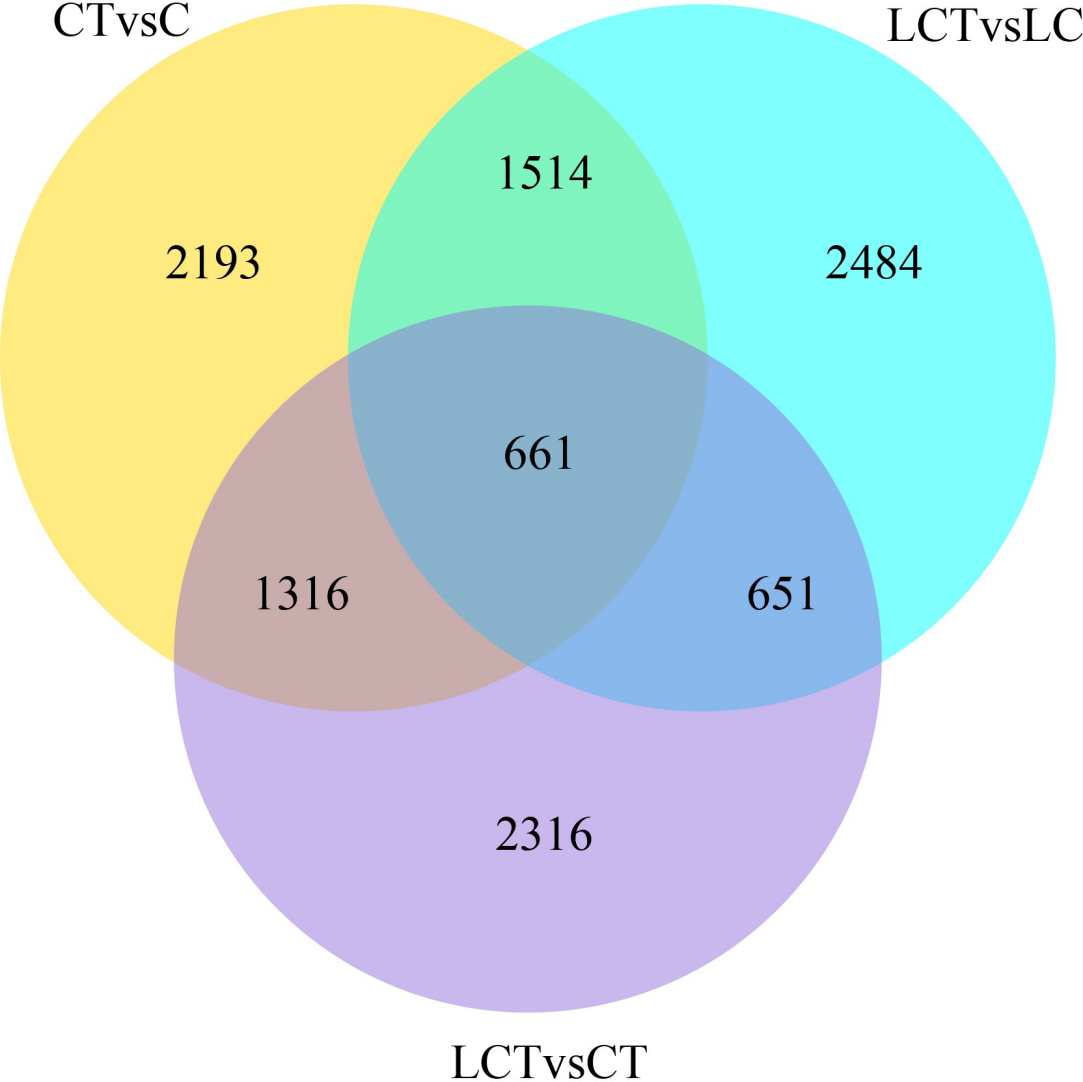
Venn diagram of differentially expressed genes.

5684, 5814, 4944, 5310, 5806 and 9831 differentially expressed genes (DEGs) were obtained in CT vs C, LCT vs C, LCT vs CT, LCT vs LC, LC vs C, and LC vs CT respectively (Fig 4). There were 3149 up-regulated and 2535 down-regulated genes in CT vs C, and 2584 up-regulated and 2726 down-regulated genes in LCT vs LC, with 2175 common DEGs between CT vs C and LCT vs LC. 1979 up-regulated and 2965 down-regulated genes were identified in LCT vs CT, and 2761 up-regulated and 3045 down-regulated genes were screened in LC vs C, with 2039 common DEGs between LCT VS CT and LC vs C. Furthermore, it is speculated that 661 shared DEGs in CT vs C, LCT vs LC and CT vs LCT may be the candidate genes for the effect of low temperature on Chinese cabbage trichomes (Fig 5).

### GO function analysis

The DEGs were annotated into 1041 GO terms which mainly distributed in structural constituent of ribosome, structural molecule activity and RNA binding in CT vs C. The DEGs screened from LCT vs LC were annotated into 1083 GO terms and mainly distributed in Structural constituent of ribosome, structural molecule activity and hydrolase activity, acting on glycosyl bonds. Furthermore, 1018 GO terms were annotated in CT vs LCT DEGs and also mainly distributed in structural constituent of ribosome, structural molecule activity and RNA binding.

**Fig 6:**
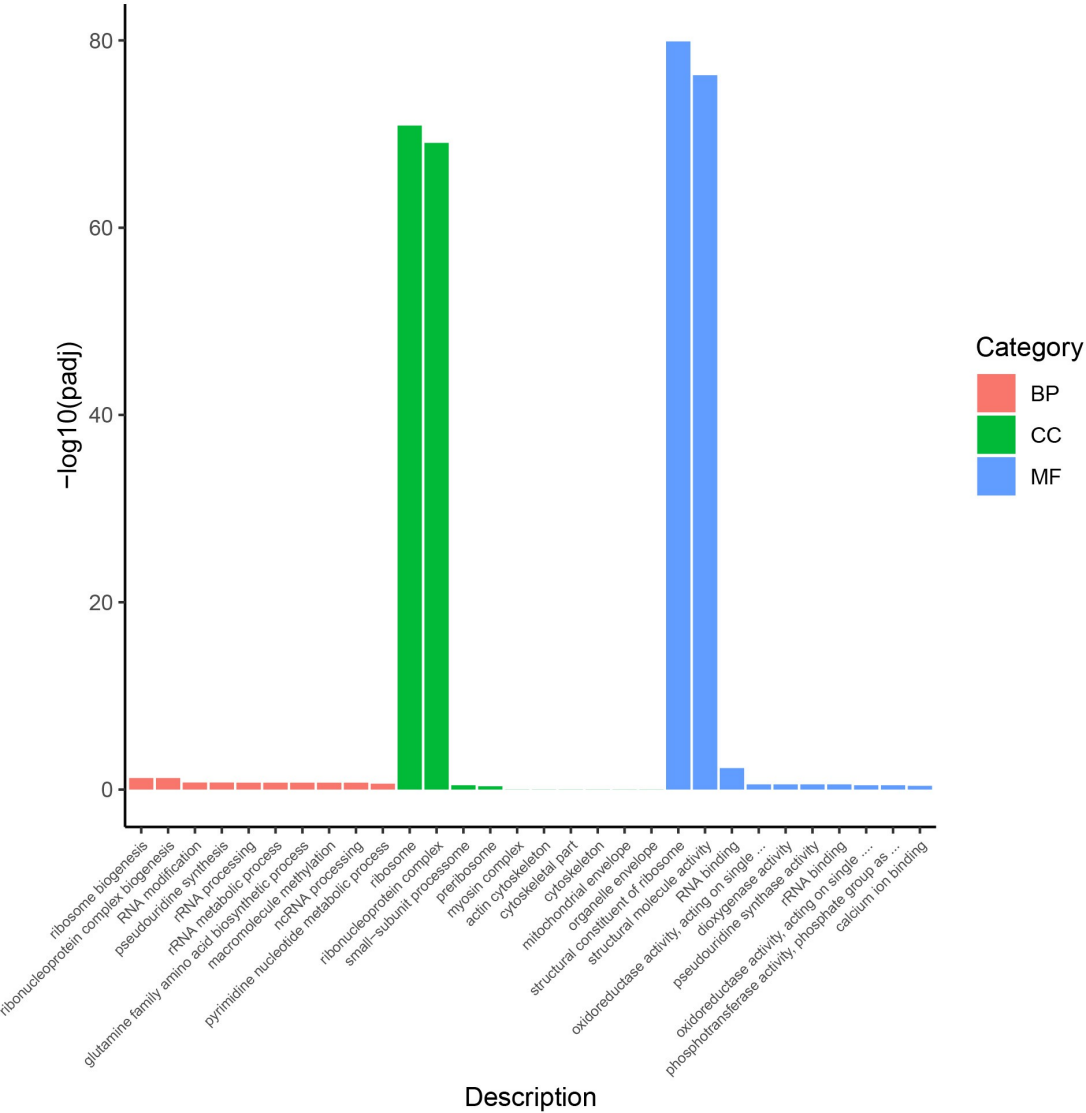
Histogram of differential gene GO enrichment.

The 661 DEGs screened out between CT vs C, LCT vs LC and CT vs LCT were annotated into 416 GO terms. Among them, the three most widely distributed differential genes in molecular function annotation are ribosomal biosynthesis, riboprotein complex and RNA modification. In the annotation of cell components, the differential genes were mainly distributed in ribosome and riboprotein complex. In the notes of biological processes, they are mainly distributed in structural components of ribosomes, structural molecular activity and RNA binding (Fig 6).

### Enrichment analysis of KEGG metabolic pathway

**Fig 7:**
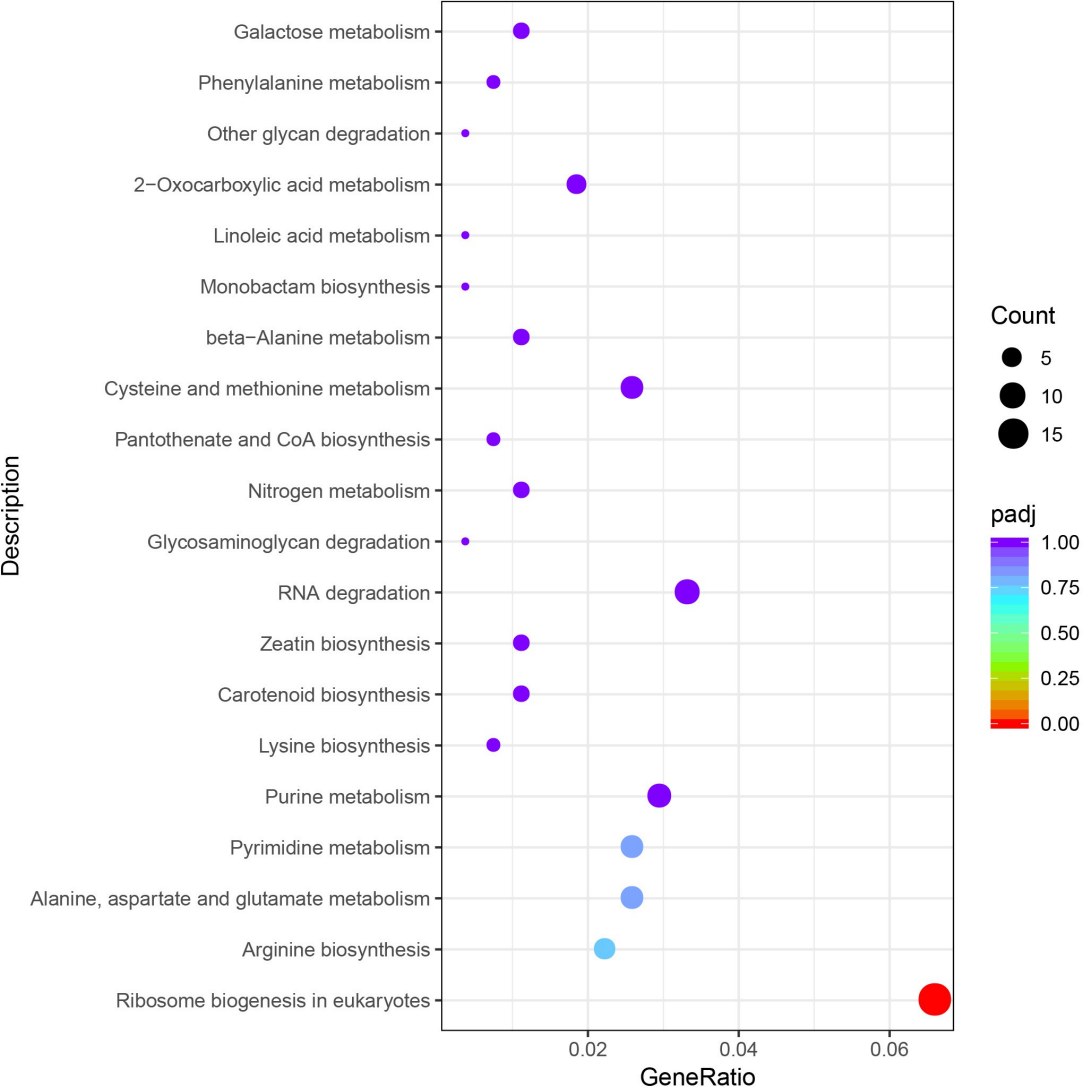
Enrichment bubble diagram of differential gene KEGG.

The enrichment results demonstrated that DEGs found in “CT vs C” were enriched in 119 metabolic pathways, among which more DEGs were enriched in plant hormone signal transduction, carbon metabolism, biosynthesis of amino acids, MAPK signaling pathway-plant pathways. And the ribosomal biogenesis and purine metabolism pathways in eukaryotes were significantly enriched. DEGs found in “LCT vs LC” were enriched in 119 metabolic pathways, with more DEGs enriched in biosynthesis of amino acid, carbon metabolism, plant hormone signal transduction, plant-pathogen interaction, 2-oxo-carbonyl acid metabolism pathways. DEGs found in “CT vs LCT” are enriched in 117 metabolic pathways, among which carbon metabolism, RNA transport, plant hormone signal transduction, ribosomal biogenesis in eukaryotes, biosynthesis of amino acid and other pathways are more enriched in DEGs. The ribosomal biogenesis in eukaryotes and photosynthesis—antenna protein pathway was significantly enriched. KEGG analysis indicated that 661 DEGs were enriched in 74 metabolic pathways, among which biosynthesis of amino acid, RNA degradation, ribosomal biogenesis in eukaryotes, carbon metabolism, purine metabolism and other pathways were more enriched. Moreover, ribosomal biogenesis in eukaryotes metabolic pathways were significantly enriched (Fig 7).

### Differential gene analysis of plant hormone signal transduction pathways and transcription factors

**Fig 8:**
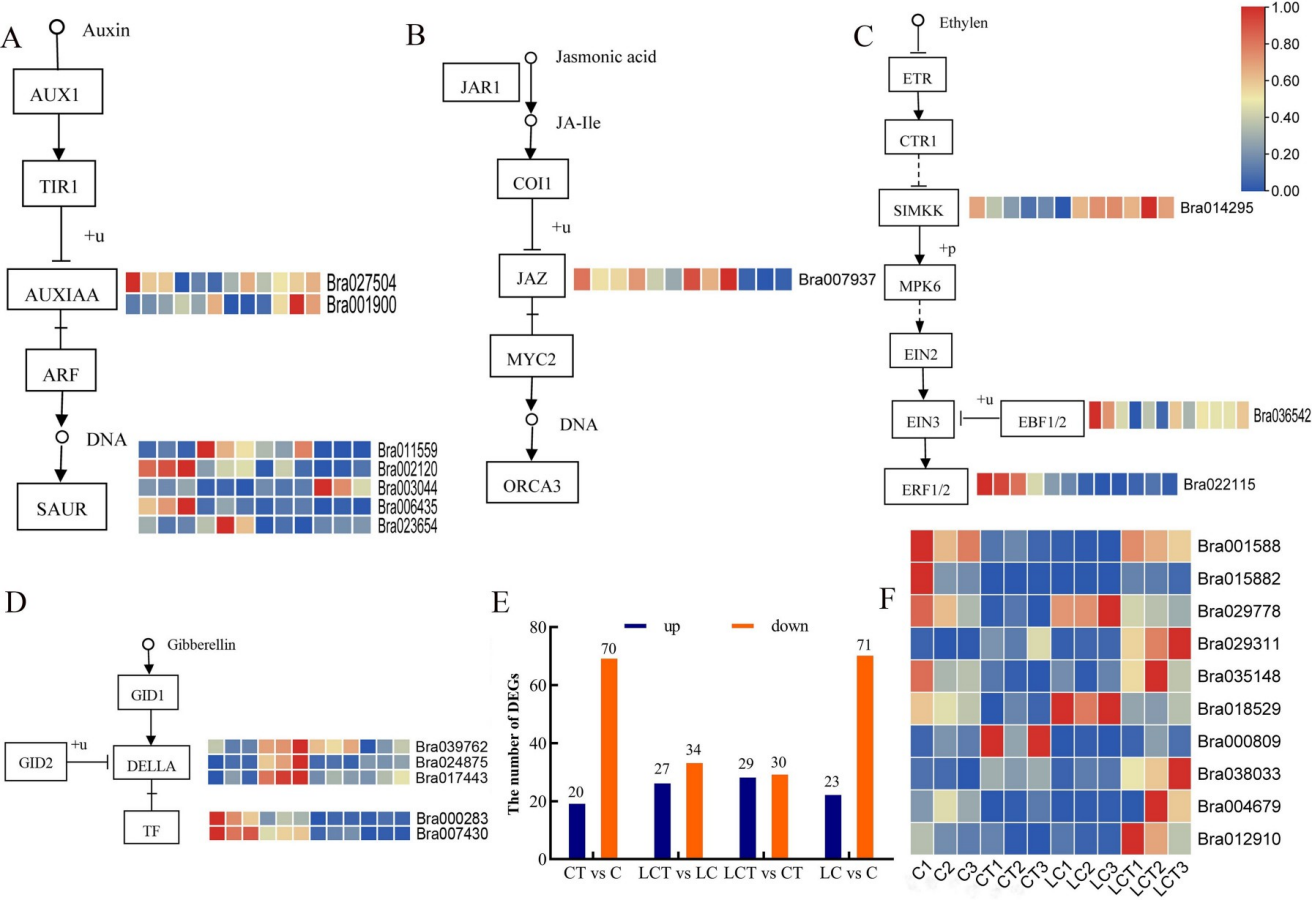
Hormone metabolic pathway and heat map of related transcription factors. A, B, C, D, Schematic diagram of gene expression related to hormone signal transduction pathway; E, Statistical histogram of 661 differential genes; F, Differential transcription factor heatmap.

According to comparison group CT vs C, LCT vs LC, CT vs LCT, KEGG metabolic pathway enrichment analysis and previous studies, the plant hormone signal transduction pathway and some significantly different transcription factors are involved in the response to the trichomes. Among the more significant difference genes, more attention was given to auxin, jasmonic acid, ethylene, gibberellin signal transduction pathway. The results showed that, 7 auxin signal transduction pathways genes including *Bra027504*, *Bra001900*, *Bra011559*, *Bra002120*, *Bra003044*, *Bra006435* and *Bra023654* were screened out of DEGs (Fig 8A), while one gene *Bra007937* appeared in jasmonic acid pathway (Fig 8B), 3 genes *Bra014295*, *Bra036542*, *Bra022115* in ethylene pathway (Fig 8C), 5 genes *Bra039762*, *Bra024875*, *Bra017443*, *Bra000283* and *Bra007430* in gibberellin pathways (Fig 8D). Furthermore, ten differentially expressed new transcription factors including *Bra001588*, *Bra015882*, *Bra029778*, *Bra029311*, *Bra035148*, *Bra018529*, *Bra000809*, *Bra038033*, *Bra004679* and *Bra012910* were obtained in 661 DEGs (Fig 8F).

### Analysis of gene weighted co-expression network among different varieties under low temperature treatment

**Fig 9:**
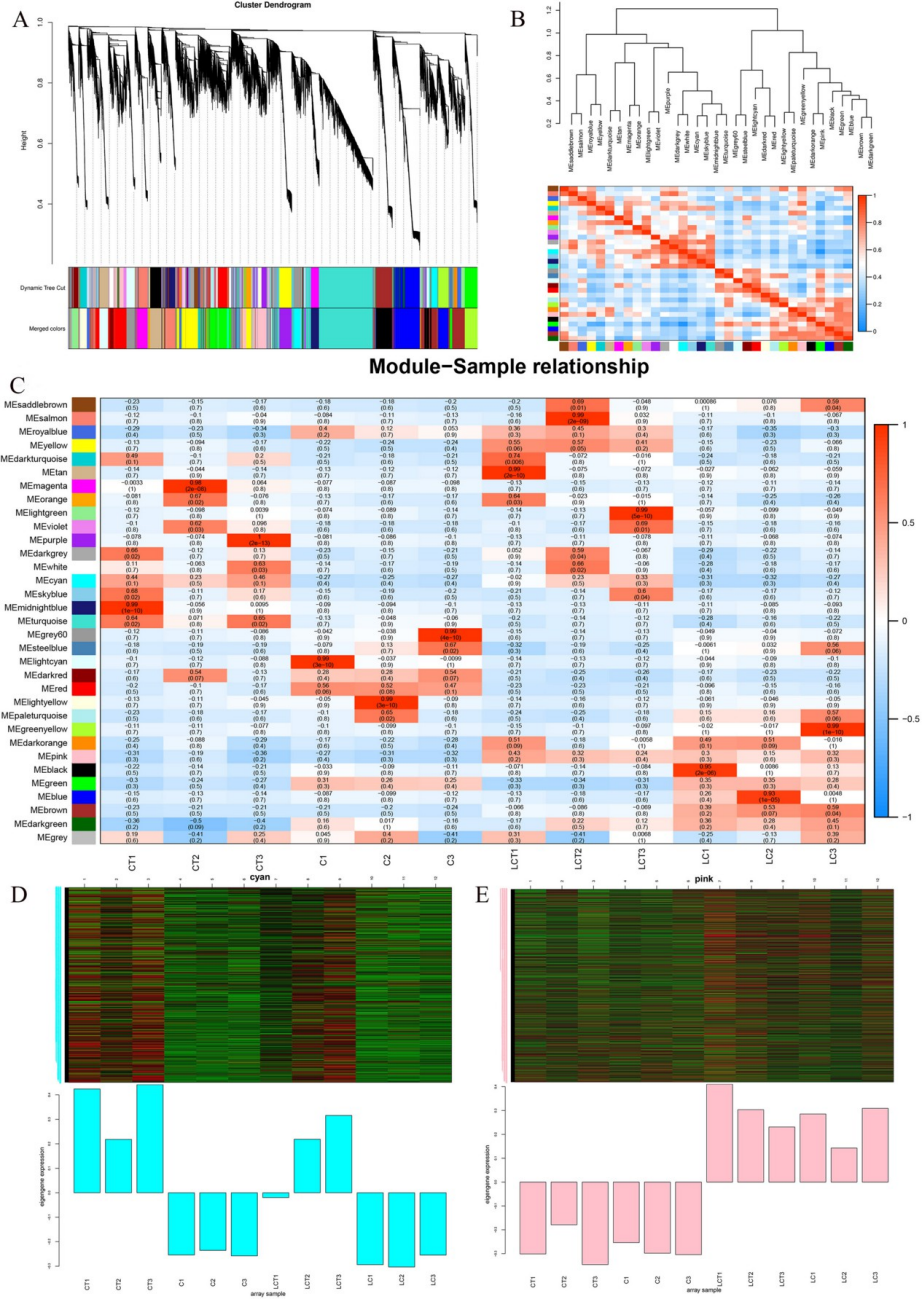
WGCNA analysis. A, Module hierarchy cluster tree diagram; B, Heat map of correlation between modules; C, Sample traits and module-to-module correlation heat map. The horizontal coordinates are samples, the ordinates are modules; D, E, Calorimetric map of gene expression in the module.

**Fig 10:**
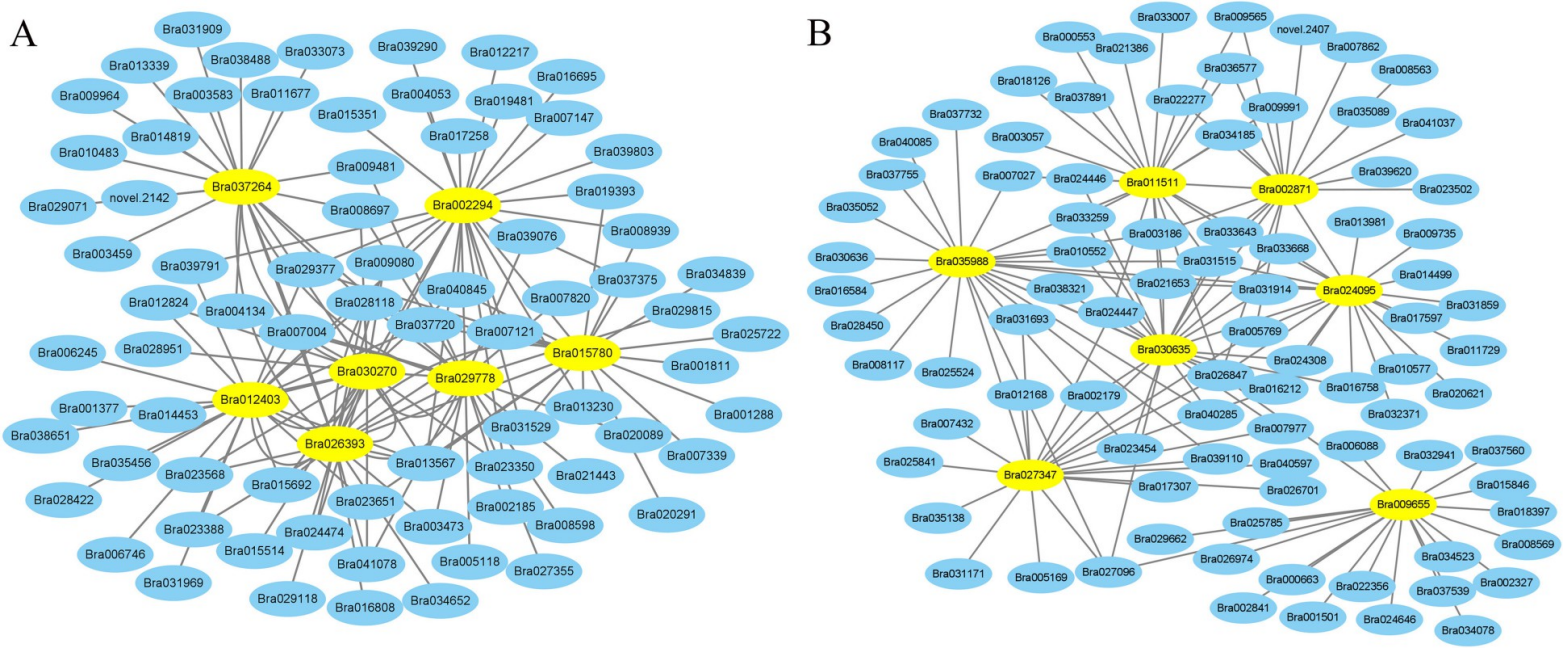
Analysis of hub gene co-expression network.

Weighted gene co-expression network analysis (WGCNA) using non-redundant DEGs was performed to investigate the regulatory network of genes related to cold treatment and trichome and 32 modules were performed (Fig 9A). This is a heat map of the correlations between modules for all genes in different modules (Fig 9B). The correlation analysis of module-sample relationship, showed that it was high correlation between modules and modules, among which cyan module is correlated with CT module and pink module is correlated with LCT module (Fig 9C). It was found that seven central hub genes *Bra029778*, *Bra037264*, *Bra002294*, *Bra030270*, *Bra012403*, *Bra026393* and *Bra015780* were noteworthy in cyan module (Figs 9D and 10A). Among the 7 highly connected central genes *Bra011511*, *Bra009655*, *Bra002871*, *Bra024095*, *Bra027347*, *Bra035988* and *Bra030635* in pink module, a related network was constructed for analysis (Figs 9E and 10B). It may speculate that these genes regulate the formation of trichomes at low temperature.

### qRT-PCR analysis of differentially expressed genes

**Fig 11:**
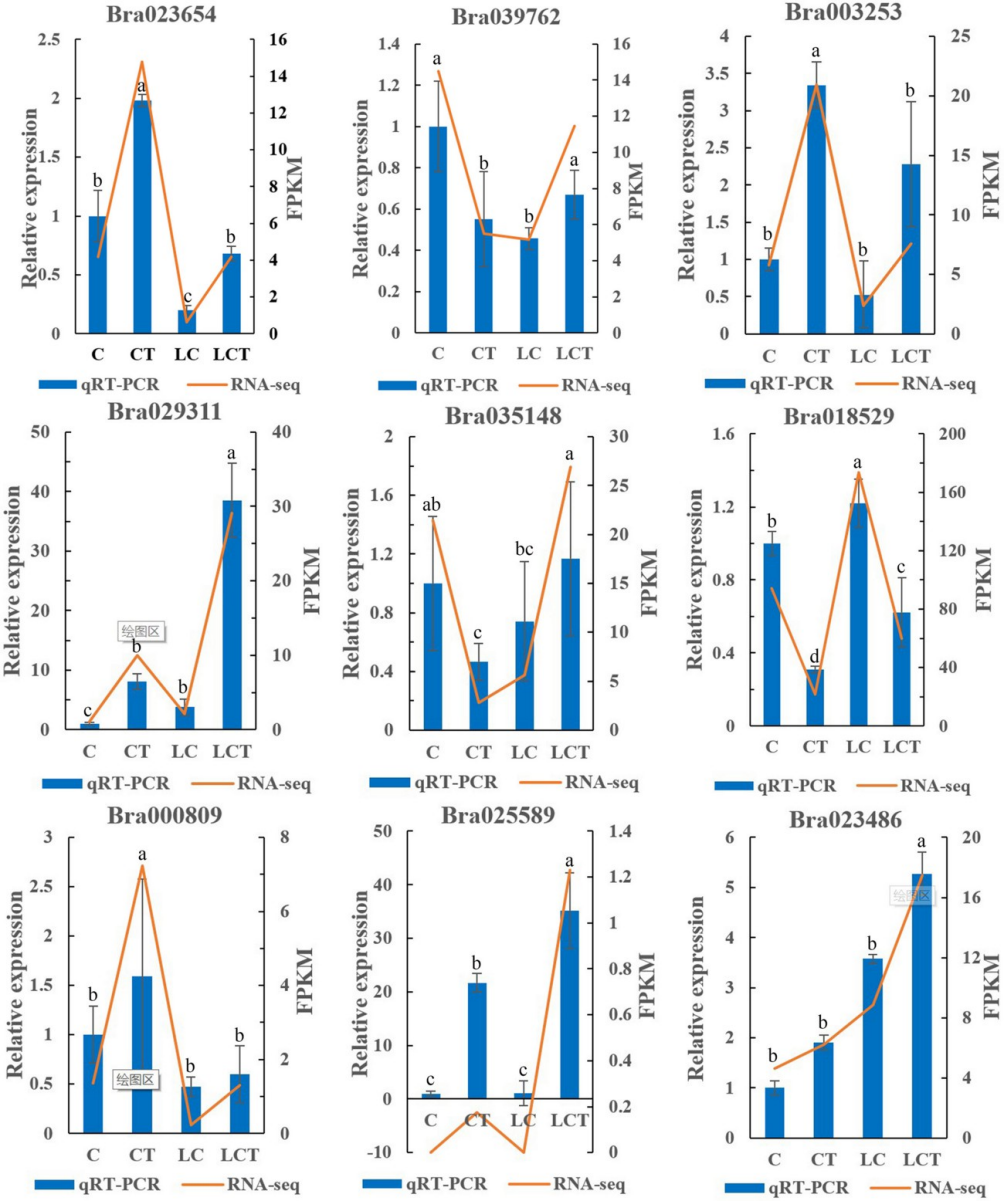
qRT-PCR verification of differential genes.

Nine DEGs closely related to the effect of low temperature on the trichomes were selected for real-time fluorescence quantitative PCR (qRT-PCR) detection (Fig 11). The qRT-PCR expression pattern is consistent with the RNA-seq trend.

## Discussion

The density of glandular hairs at young leaves, stem tips and flower buds of many plants was higher than that of mature leaves, stems and flowers [11, 33], and the glandular hair density of the same leaf at different positions of front and back, edge and center was also different, especially represented by tomato and *Artemisia annua*. The external environment has a certain influence on the trichomes of Chinese cabbage. Temperature controls the phenotype by regulating the balance of various metabolic pathways in plants. Previous study reported that the differential DEGs of trichomes were mainly enriched in primary and secondary metabolic biosynthesis pathways [32]. In this study, we found that the number of trichomes on the adaxial, abaxial and edge of leaves was different, there were more trichomes on the abaxial and edge of leaves, which were 2.2 and 2.6 times of that on the adaxial, respectively. Compared with low temperature treatment, the DEGs from three comparison groups were mainly enriched in plant hormone signal transduction and biosynthesis of amino acids pathways. Furthermore, the differential genes were significantly up or down-regulated, and these genes can be involved in trichome formation.

Biosynthesis and signal transduction pathways of plant hormones are involved in the development of trichome [34]. Transcriptome analysis showed that auxin, ethylene and cytokinin regulate a set of similar root hair specific genes that control root hair elongation in *Arabidopsis thaliana*. Gibberellin signal transduction controls the accumulation and expression of gibberellin biosynthesis genes to regulate positive regulation of tobacco glandular hair initiation [35]. Spraying gibberellin on the surface of flue-cured tobacco increases the density of leaf glandular hairs [36]. Methyl jasmonate is a signal transduction molecule in plants, and exogenous application significantly improved the transcription level of key genes in *Camptotheca acuminata* coat synthesis and promoted the significant increase of *Camptotheca acuminata* coat density [37]. However, it has been reported that plant hormones are not directly involved in regulating trichome development, but indirectly regulate by the expression levels of positive or negative regulatory factors [38]. We identified the hormone metabolism pathways were enriched before and after low temperature treatment. At room temperature, 90 DEGs were enriched in the hormone signal transduction pathway of Chinese cabbage with or without trichomes, including 20 up-regulated DEGs and 70 down-regulated DEGs. After low temperature treatment, 105 DEGs were enriched in plant hormone signal transduction pathway, among which 28 DEGs were up-regulated and 77 DEGs were down-regulated. Compared with the trichomes before and after treatment, 59 DEGs were enriched in plant hormone signal transduction pathways, among which 29 DEGs were up-regulated and 30 DEGs were down-regulated. Therefore, this experiment further proved the relationship between burrs and hormones, and screened the hormone genes *Bra039762*, *Bra007937*, *Bra017443*, *Bra24875*, *Bra027504*, *Bra023654* that may affect the formation of trichomes, laying a foundation for improving the molecular mechanism of hormone regulation of trichomes.

Transcription factors are important regulatory factors widely existing, and play a significant role in the regulation of trichome development. R2R3-MYB transcription factor and bHLH-like transcription factor can jointly form a trimer complex activator MYB-bHLH-WD40 [39, 40], directly acting on GL2/TTG2, positively regulates the development of *Arabidopsis* trichome [41, 42]. It has been found that R2R3-MYB transcription factor genes, bHLH transcription factors and HD-ZIP IV transcription factor genes are involved in the regulation of plant surface trichome development [43–49]. In addition, the AP2/ERF transcription factor OsHL6 protein interacts with OsWOX3B, a key regulator of rice surface coat initiation, to promote surface coat initiation and elongation [50]. Some WRKY, ERF and bZIP transcription factors can be specifically expressed in glandular hairs, and different types interact with each other. *Bra001588*, *Bra015882*, *Bra029778*, *Bra029311*, *Bra035148*, *Bra018529*, *Bra000809*, *Bra038033*, *Bra004679* and *Bra012910* were screened according to expression level and CT vs C, LCT vs LC and CT vs LCT comparison group. The 10 transcription factors are related to the effect of low temperature on the trichomes. Among them, Bra029778 gene was also screened in the co-expression network, which is the central hub gene and can be verified for subsequent function. It can be seen that the regulation of transcriptional interaction induced by low temperature in plant trichome formation needs to be further explored.

Plant trichomes often secrete and synthesize different types of defense substances, such as terpenoids, amino acids, phenylpropanes, lipid derivatives. These secondary metabolites can protect plants from biological and abiotic stress and play an important role in defense. Phenylalanine, tryptophan and tyrosine are all aromatic amino acids, among which tryptophan is the precursor of auxin, alkaloids, indoline and plant antitoxin, while tyrosine is the precursor of isoquinoline alkaloids, betaine and quinones [51, 52], phenylalanine is the precursor of phenylcyclopropane pathway, and p-coumaryl coenzyme A is the intermediate of phenyl-propane metabolic pathway, as well as the precursor of various substances such as phenyl-propylene and flavonoids [53]. The amino acid biosynthesis pathway of Chinese cabbage with or without trichomes was enriched in 88 DEGs, including 59 up-regulated DEGs and 29 down-regulated DEGs. After low temperature treatment, 76 DEGs were enriched in amino acid biosynthesis pathway, among which 30 DEGs were up-regulated and 46 DEGs were down-regulated. Compared with trichomes before and after treatment, 78 DEGs were enriched in amino acid biosynthesis pathway, 13 DEGs were up-regulated and 65 DEGs were down-regulated. *Bra026393*, *Bra030270*, *Bra037264*, *Bra009655*, *Bra019206*, *Bra021682*, *Bra020605*, *Bra040146*, *Bra016680* may be related to the effect of low temperature on trichomes. *Bra026393*, *Bra030270*, *Bra037264* and *Bra009655* were also selected in the gene co-expression network as the central hub genes.

Hormone signal transduction pathway and amino acid biosynthesis pathway were obtained by KEGG enrichment, and 661 differential genes were enriched in the three comparison groups. The differential genes were screened for qRT-PCR analysis. *Bra029311*, *Bra035148*, *Bra018529*, *Bra000809*, *Bra003253*, *Bra025589* and *Bra023486* were selected transcription factors in 661 genes. *Bra039762* and *Bra023654* were screened genes in plant hormone signal transduction pathway. The results were consistent with the transcriptome trend, which proved the reliability of transcriptome data. The expression levels of 9 genes were significantly different between trichome Chinese cabbage CT and without trichome Chinese cabbage C. After low temperature treatment, the expression level increased or decreased, and the difference was significant. It may be related to the effect of low temperature on the trichomes. The genes enriched in related pathway and comparison group were combined with WGCNA for analysis, and five genes *Bra029778*, *Bra026393*, *Bra030270*, *Bra037264* and *Bra009655* were screened together. The reliability was strong, and the subsequent functional verification could be carried out. In order to study the phenotypic changes and molecular mechanism of Chinese cabbage leaf trichomes in response to low temperature, a new path was explored.

## References

[1] Balkunde R, Pesch M, Hulskamp M. Trichome patterning in *Arabidopsis thaliana*: From genetic to molecular models. Current Topics in Developmental Biology. 2010;91: 299–321. 20705186 10.1016/S0070-2153(10)91010-7

[2] Huang CZ, Jiao XM, Yang L, Zhang MM, Dai MM, Wang L, et al. ROP-GEF signal transduction is involved in At CAP_1_-regulated root hair growth. Plant Growth Regulation. 2019a;87 (1): 1–8.

[3] Andrade M C, Da Silva A, Neiva I P, Oliveira I R, Da Silva E, David M F, et al. Inheritance of type IV glandular trichome density and its association with whitefly resistance from *Solanum galapagense* accession LA1401. Euphytica. 2017;213 (52): 1–12.

[4] Heil M. Indirect defence via tritrophic interactions. New Phytologist. 2010;178 (1): 41–61. 10.1111/j.1469-8137.2007.02330.x 18086230

[5] Li C, Wang Z, Jones AD. Chemical imaging of trichome specialized metabolites using contact printing and laser desorption/ionization mass spectrometry. Analytical and Bioanalytical Chemistry. 2014;406(1): 171–182. doi: 10.1007/s00216-013-7444-6 24220760

[6] Weinhold A, Baldwin IT. Trichome-derived O-acyl sugars are a first meal for caterpillars that tags them for predation. Proceedings of the National Academy of Sciences of United States of America. 2011;108 (19): 7855–7859. doi: 10.1073/pnas.1101306108 21518882 PMC3093468

[7] Gao W, Zhou Y, Wang X, Jiao H, Haibo L, Miao HE. Effects of UV-B radiation on the glandular trichomes and photosynthetic characteristics of *Dendranthema indicum* var. *aromaticum*. Journal of Hunan Agricultural University. 2016.

[8] Li K, Li SJ, Zhou ZY, Yao HZ, Zhou Y, Tang XQ, et al. Effects of drought stress on glandular trichome density and volatile exudates of *Schizonepeta tenuifolia*. China Journal of Chinese Materia Medica. 2019;44(21): 4573–4580. 31872650 10.19540/j.cnki.cjcmm.20190829.104

[9] Yang C, Ye Z. Trichomes as models for studying plant cell differentiation. Cellular and Molecular Life Sciences. 2013;70 (11): 1937–1948. doi: 10.1007/s00018-012-1147-6 22996257 PMC11113616

[10] Zhou Y, Tang N, Huang L, Zhao Y, Tang X, Wang K. Effects of salt stress on plant growth, antioxidant capacity, glandular trichome density, and volatile exudates of *Schizonepeta tenuifoliabriq*. International Journal of Molecular Sciences. 2018;19(1):1–15. 10.3390/ijms19010252 PMC5796199 29342961

[11] Lihavainen J, Ahonen V, Keski-Saari S, Sõber A, Oksanen E, Keinänen M. Low vapor pressure deficit reduces glandular trichome density and modifies the chemical composition of cuticular waxes in silver birch leaves. Tree Physiology. 2017;37(9): 1166–1181. doi: 10.1093/treephys/tpx045 28460081

[12] Bryant L, Patole C, Cramer R. Proteomic analysis of the medicinal plant *Artemisia annua*: data from leaf and trichome extracts. Data in Brief. 2016;7:325–331. 26977431 10.1016/j.dib.2016.02.038 PMC4781977

[13] Tattini M, Gravano E, Pinelli P, Mulinacci N, Romani A. Flavonoids accumulate in leaves and glandular trichomes of *Phillyrea latifolia* exposed to excess solar radiation. New Phytologist. 2000;148 (1): 69–77. 33863030 10.1046/j.1469-8137.2000.00743.x

[14] Han G, Li Y, Yang Z, Wang C, Zhang Y, Wang B. Molecular mechanism of plant trichome development. Frontiers in Plant Science. 2022; 13:1–26. 10.3389/fpls.2022.910228 PMC9198495 35720574

[15] Werker E. Trichome diversity and development. Advances in Botanical Research. 2000;31: 1–35.

[16] Schollert M, Kivimäenpää M, Michelsen A, Blok D, Rinnan R. Leaf anatomy, BVOC emission and CO_2_ exchange of arctic plants following snow addition and summer warming. Annals of Botany. 2017;119: 433–445. 28064192 10.1093/aob/mcw237 PMC5314650

[17] Giuliani C, Maggi F, Papa F, Maleci Bini L. Congruence of phytochemical and morphological profiles along an altitudinal gradient in *Origanum vulgare* ssp. vulgare from Venetian region (NE Italy). Chemistry Biodiversity. 2013;10 (4): 569–583. 23576343 10.1002/cbdv.201300019

[18] Liu JQ, Chen K, Zhang ZZ, Chen XL, Wang AX. Effects of exogenous GA, MeJA, IAA, SA and KT on trichome formation in tomato. Journal of Horticulture.2016;43(11): 2151–2160.

[19] Li C, Wang P, Menzies N W, Lombi E, Kopittke PM. Effects of methyl jasmonate on plant growth and leaf properties. Journal of Plant Nutrition and Soil Science. 2018;181(3): 409–418.

[20] Kazama H, Dan H, Imaseki H, Wasteneys GO. Transient exposure to ethylene stimulates cell division and alters the fate and polarity of hypocotyl epidermal cells. Plant Physiology. 2004;134 (4): 1614–1623. doi: 10.1104/pp.103.031088 15047904 PMC419835

[21] Wasternack C, Hause B. Jasmonates: biosynthesis, perception, signal transduction and action in plant stress response, growth and development. Annals of Botany. 2013;111(6): 1021–58. 23558912 10.1093/aob/mct067 PMC3662512

[22] Wasternack C, Strnad M. Jasmonates are signals in the biosynthesis of secondary metabolites—Pathways, transcription factors and applied aspects–A brief review. New Biotechnology. 2017;48: 1–11. doi: 10.1016/j.nbt.2017.09.007 29017819

[23] Huang H, Liu B, Liu L, Song S. Jasmonate action in plant growth and development. Journal of Experimental Botany. 2017;68(6): 1349–1359. doi: 10.1093/jxb/erw495 28158849

[24] Kirik V, Schnittger A, Radchuk V, Adler K, Hülskamp M, Bäumlein H. Ectopic expression of the *Arabidopsis AtMYB23* gene induces differentiation of trichome cells. Developmental Biology. 2001;235, 366–377. 11437443 10.1006/dbio.2001.0287

[25] Kirik V, Grini PE, Mathur J, Klinkhammer I, Adler K, Bechtold N, et al. The *Arabidopsis* TUBULIN-FOLDING COFACTOR A gene is involved in the control of the alpha/beta-tubulin monomer balance. Plant Cell. 2002;14, 2265–2276. 12215519 10.1105/tpc.003020 PMC150769

[26] Kirik V, Lee MM, Wester K, Herrmann U, Zheng Z, Oppenheimer D, et al. Functional diversification of *MYB23* and *GL1* genes in trichome morphogenesis and initiation. Development. 2005;132(7): 1477–1485. 15728674 10.1242/dev.01708

[27] Gan Y, Liu C, Yu H, Broun P. Integration of cytokinin and gibberellin signaling by *Arabidopsis* transcription factors GIS, ZFP8 and GIS2 in the regulation of epidermal cell fate. Development. 2007;134: 2073–2081. 17507408 10.1242/dev.005017

[28] Li F, Kitashiba H, Inaba K, Nishio T. A *Brassica rapa* linkage map of EST-based SNP markers for identification of candidate genes controlling flowering time and leaf morphological traits. DNA Research. 2009;16(6):311–323. 19884167 10.1093/dnares/dsp020 PMC2780953

[29] Li F, Kitashiba H, Nishio T. Association of sequence variation in *Brassica GLABRA*_*1*_ orthologs with leaf hairiness. Molecular Breeding. 2011;28(4):577–584.

[30] Zhang J, Lu Y, Yuan Y, Zhang X, Geng J, Chen Y, et al. Map-based cloning and characterization of a gene controlling hairiness and seed coat color traits in *Brassica rapa*. Plant Molecular Biology. 2009; 69(5):553–563. 19039665 10.1007/s11103-008-9437-y

[31] Kawakatsu Y, Nakayama H, Kaminoyama K, Igarashi K, Yasugi M, Kudoh H, et al. A GLABRA1 ortholog on LG A9 controls trichome number in the Japanese leafy vegetables Mizuna and Mibuna (Brassica rapa L. subsp. nipposinica L. H. Bailey): evidence from QTL analysis. Journal of Plant Research. 2017;130(3):539–550. doi: 10.1007/s10265-017-0917-5 28258381

[32] Yan T, Chen M, Shen Q, Li L, Fu X, Pan Q, et al. Homeodomain protein 1 is required for jasmonate-mediated glandular trichome initiation in *Artemisia annua*. New Phytologist, 2017, 213(3): 1145–1155. 27659595 10.1111/nph.14205

[33] Wang HX. Identification of yield related QTLs and fine mapping and gene cloning of leaf trichome in Chinese cabbage. Shandong University. 2018.

[34] Pattanaik S, Patra B, Singh SK, Yuan L. An overview of the gene regulatory network controlling trichome development in the model plant, *Arabidopsis*. Frontiers in Plant Science. 2014;5: 1–8. 10.3389/fpls.2014.00259 PMC4071814 25018756

[35] Liu Y, Liu D, Khan A R, Liu B, Wu M, Huang L, et al. NbGIS regulates glandular trichome initiation through GA signaling in tobacco. Plant Molecular Biology. 2018; 98:153–167. doi: 10.1007/s11103-018-0772-3 30171399

[36] He LX, Xue G, Sun JT, Zhang ZQ, Ding YL, Yang TL, et al. Effects of exogenous gibberellin on flue-cured tobacco leaf trichome and aromatic substances. Journal of Henan Agricultural Sciences. 2021;50(01): 52–59.

[37] Ma WH, Wang YY, Yu F. Exogenous methyl jasmonate regulates the trichome development and camptothecin biosynthesis of *Camptothecin acuminata* leaves. Molecular Plant Breeding. 2021;1–9.

[38] Liu XM, Tang N, Chen ZX, Luo CR, Zhang W, Xu F. Progress in plant trichome development research. Acta Horticulturae Sinica. 2021;48(04): 705–718.

[39] Zhao M, Morohashi K, Hatlestad G, Grotewold E, Lloyd A. The TTG1-bHLH-MYB complex controls trichome cell fate and patterning through direct targeting of regulatory loci. Development. 2008;135(11): 1991–1999. doi: 10.1242/dev.016873 18434419

[40] Pesch M, Schultheib I, Klopffleisch K, Uhrig JF, Koegl M, Clemen CS, et al. Transparent Testa GLABRA1 and GLABRA1 compete for binding to GLABRA3 in *Arabidopsis*. Plant Physiology. 2015;168(2):584–597. 25926482 10.1104/pp.15.00328 PMC4453790

[41] Rerie WG, Feldmann KA, Marks MD. The GLABRA2 gene encodes a homeo domain protein required for normal trichome development in *Arabidopsis*. Genes Development. 1994;8(12): 1388–1399. 7926739 10.1101/gad.8.12.1388

[42] Wang SC, Chen JG. Arabidopsis transient expression analysis reveals that activation of GLABRA2 may require concurrent binding of GLABRA1 and GLABRA3 to the promoter of GLABRA2. Plant Cell Physiology. 2008;49(12): 1792–1804. doi: 10.1093/pcp/pcn159 18948276

[43] Oppenheimer DG, Herman PL, Sivakumaran S, Esch J, Marks MD, et al. A myb gene required for leaf trichome differentiation in *Arabidopsis* is expressed in stipules. Cell. 1991;67(3):483–493. 1934056 10.1016/0092-8674(91)90523-2

[44] Schnittger A, Folkers U, Schwab B, Jürgens G, Hülskamp M. Generation of a spacing pattern: the role of triptychon in trichome patterning in *Arabidopsis*. Plant Cell. 1999;11 (11):1105–16. 10368181 10.1105/tpc.11.6.1105 PMC144244

[45] Schellmann S, Schnittger A, Kirik V, Wada T, Okada K, Beermann A, et al. TRIPTYCHON and CAPRICE mediate lateral inhibition during trichome and root hair patterning in *Arabidopsis*. EMBO Journal. 2002;21(19):5036–5046. 12356720 10.1093/emboj/cdf524 PMC129046

[46] Payne CT, Zhang F, Lloyd AM. GL3 encodes a bHLH protein that regulates trichome development in *Arabidopsis* through interaction with GL1 and TTG1. Genetics. 2000;156(3):1349–1362. 11063707 10.1093/genetics/156.3.1349 PMC1461316

[47] Zhang F, Gonzalez A, Zhao M, Payne CT, Lloyd A. A network of redundant bHLH proteins functions in all TTG1-dependent pathways of *Arabidopsis*. Development. 2003;130(5):4859–4869. 12917293 10.1242/dev.00681

[48] Galway ME, Masucci JD, Lloyd AM, Walbot V, Davis RW, Schiefelbein JW. The *TTG1* gene is required to specify epidermal cell fate and cell patterning in the *Arabidopsis* root. Developmental Biology. 1994;166(2):740–754. 7813791 10.1006/dbio.1994.1352

[49] Chini A, Gimenez-Ibanez S, Goossens A, Solano R. Redundancy and specificity in jasmonate signaling. Current Opinion in Plant Biology. 2016;33: 147–156. 27490895 10.1016/j.pbi.2016.07.005

[50] Sun WQ, Gao DW, Xiong Y, Tang X, Xiao X, Wang C, et al. Hairy Leaf 6, an AP2/ERF transcription factor, interacts with OsWOX3B and regulates trichome formation in rice. Molecular Plant, 2017, 10(11): 1417–1433. doi: 10.1016/j.molp.2017.09.015 28965833

[51] Maeda H, Dudareva N. The shikimate pathway and aromatic amino acid biosynthesis in plants. Annual Review of Plant Biology. 2012;63: 73–105. doi: 10.1146/annurev-arplant-042811-105439 22554242

[52] Barros J, Serrani–Yarce JC, Chen F, Baxter D, Venables BJ, Dixon RA. Role of bifunctional ammonialyase in grass cell wall biosynthesis. Nature Plants. 2016;2(6):16050. doi: 10.1038/nplants.2016.50 27255834

[53] Vogt T. Phenylpropanoid biosythesis. Molecular Plant. 2010;3(1):2–20. 20035037 10.1093/mp/ssp106

