# Supplementary material for: Insight into the effect of low temperature treatment on trichome density and related differentially expressed genes in Chinese cabbage
Source: PLoS One. 2022 Sep 15;17(9):e0274530. doi: 10.1371/journal.pone.0274530 (PMC9477275; doi:10.1371/journal.pone.0274530)
Supplement: Attachment — Submitted filename: Response to Reviewers.docx [file pone.0274530.s001.docx]

Dear Academic Editors and reviewers,

I appreciate the comments on my paper from Academic Editors and reviewers very much. According to your opinions, I have carefully revised my paper again. Please see the revised draft for details. The answers to specific questions are as follows:

Question：During our internal evaluation of the manuscript, we found significant text overlap between your submission and previous work in the [introduction, conclusion, etc.].

The introduction, conclusion etc are revised further.

Reply to Reviewer 1:

Question 1: The English writing is very difficult to understand such as ‘Now, all Raw dates obtained by sequencing have been submitted to NCBI, login number: PRJNA842790.’ which might be ‘Raw data’. Therefore, the manuscript needs native English editing.

The English language expression and format have been further modified.

Question 2: The references are not match with the citation in MS correctly, such as ‘Chinese cabbage trichomes play an obvious role in increasing leaf thickness to reduce heat of epidermis [26], loss of water [27], and defense against insect pathogen invasion and mechanical injury [28-29], but it also affects the consumption quality of cabbage.’ The references of 26 and 27 the authors listed are not mentioned about Chinese cabbage trichomes at all. Check it please.

The two references cited have been removed.

Question 3: The authors need describe the materials and methods clearly, such as ‘CT and C are treated at room temperature, while LCT and LC are treated at low temperature.’ What are LCT and LC exactly? Moreover, give the numbers of seedlings in each treatment instead of ‘some of’ and ‘the remaining’.

It has been revised. See the revised manuscript for details.

Question 4: ‘After 10 days of treatment at low and normal temperature, 3 cabbage plants were taken from each treatment and 3 leaves were selected from top to bottom.’ Pay attention to Chinese cabbage and cabbage which are totally different.

It is revised to Chinese cabbage.

Question 5: Table 1 need remove to results part.

The primers were placed in the experimental methods section in most articles.

Question 6: The authors need rewrite the table and figure legends clearly.

These have been revised, please see the revised manuscript that will be uploaded.

Question 7: The most important data analysis process of the final selected 9 genes for qRT-PCR validation is not mentioned in results part.

qRT-PCR analysis has been added to conclusions and results. Detailed in manuscript 257-259 and 332-346.

Question 8: The abstract and conclusion need reorganize carefully.

The abstract and conclusion have been greatly revised.

Reply to Reviewer 2:

Question 1: The authors focused on the DEGs derived from comparison groups of ‘CT vs C, LCT vs LC and CT vs LCT’, while the underlying biological significances should be addressed and explained in this MS.

It has been revised. See the revised manuscript for details.

Question 2: I did not find the result descriptions of Fig 10., looks like this part was missed in Results part or perhaps this is not a necessary Fig to this MS.

Fig 10 analysis has been added to conclusions and results. Detailed in manuscript 242-247.

Question 3: Fig 11. should include the statistically significant analysis since these 9 genes are significant DEGs selected from RNA-seq data. The ‘Conclusion’ part actually is the ‘Discussion’, please modify accordingly and add the proper conclusion of this MS.

Fig 11 analysis has been added to conclusions. Detailed in manuscript 257-259 and 332-346.

Question 4: In this MS, the low temperature treatment also affected the Chinese cabbage plant growth (size), how to distinguish the DEGs are truly related with trichome density regulation rather than involving in plant growth (size) or growth speed (slow or quick). Besides, this manuscript needs further editing for language and writing quality.

Two reasons for DEGs selection as follows. The first is the comparison between with and without trichome Chinese cabbage, and between room temperature and low temperature, which does not involve the development stage. Secondly, the correlation between trichome and gene expression was analyzed by WGCNA.

I appreciate the reviewer's comments and accept them. Thank you very much for your review.
