# Supplementary material for: Insight into the effect of low temperature treatment on trichome density and related differentially expressed genes in Chinese cabbage
Source: PLoS One. 2022 Sep 15;17(9):e0274530. doi: 10.1371/journal.pone.0274530 (PMC9477275; doi:10.1371/journal.pone.0274530)
Supplement: Peer review history

# Peer review: Decision Letter 0

**DOI:** 10.1371/journal.pone.0274530.r001

**Authors:** Maoteng Li

11 Aug 2022

PONE-D-22-18944Insight into the Effect of Low Temperature Treatment on Trichome Density and Related Differentially Expressed Genes in Chinese CabbagePLOS ONE

Dear Dr. Cheng,

Thank you for submitting your manuscript to PLOS ONE. After careful consideration, we feel that it has merit but does not fully meet PLOS ONE’s publication criteria as it currently stands. Therefore, we invite you to submit a revised version of the manuscript that addresses the points raised during the review process.

Please submit your revised manuscript by Sep 25 2022 11:59PM. If you will need more time than this to complete your revisions, please reply to this message or contact the journal office at plosone@plos.org. When you're ready to submit your revision, log on to https://www.editorialmanager.com/pone/ and select the 'Submissions Needing Revision' folder to locate your manuscript file.

Please include the following items when submitting your revised manuscript:

- A rebuttal letter that responds to each point raised by the academic editor and reviewer(s). You should upload this letter as a separate file labeled 'Response to Reviewers'.
- A marked-up copy of your manuscript that highlights changes made to the original version. You should upload this as a separate file labeled 'Revised Manuscript with Track Changes'.
- An unmarked version of your revised paper without tracked changes. You should upload this as a separate file labeled 'Manuscript'.

If you would like to make changes to your financial disclosure, please include your updated statement in your cover letter. Guidelines for resubmitting your figure files are available below the reviewer comments at the end of this letter.

If applicable, we recommend that you deposit your laboratory protocols in protocols.io to enhance the reproducibility of your results. Protocols.io assigns your protocol its own identifier (DOI) so that it can be cited independently in the future. For instructions see: https://journals.plos.org/plosone/s/submission-guidelines#loc-laboratory-protocols. Additionally, PLOS ONE offers an option for publishing peer-reviewed Lab Protocol articles, which describe protocols hosted on protocols.io. Read more information on sharing protocols at https://plos.org/protocols?utm_medium=editorial-email&utm_source=authorletters&utm_campaign=protocols.

We look forward to receiving your revised manuscript.

Kind regards,

Maoteng Li

Academic Editor

PLOS ONE

Journal Requirements:

When submitting your revision, we need you to address these additional requirements.

1. Please ensure that your manuscript meets PLOS ONE's style requirements, including those for file naming. The PLOS ONE style templates can be found at 

https://journals.plos.org/plosone/s/file?id=wjVg/PLOSOne_formatting_sample_main_body.pdf and 

https://journals.plos.org/plosone/s/file?id=ba62/PLOSOne_formatting_sample_title_authors_affiliations.pdf

2. Thank you for submitting the above manuscript to PLOS ONE. During our internal evaluation of the manuscript, we found significant text overlap between your submission and previous work in the [introduction, conclusion, etc.].

We would like to make you aware that copying extracts from previous publications, especially outside the methods section, word-for-word is unacceptable. In addition, the reproduction of text from published reports has implications for the copyright that may apply to the publications.

Please revise the manuscript to rephrase the duplicated text, cite your sources, and provide details as to how the current manuscript advances on previous work. Please note that further consideration is dependent on the submission of a manuscript that addresses these concerns about the overlap in text with published work.

[If the overlap is with the authors’ own works: Moreover, upon submission, authors must confirm that the manuscript, or any related manuscript, is not currently under consideration or accepted elsewhere. If related work has been submitted to PLOS ONE or elsewhere, authors must include a copy with the submitted article. Reviewers will be asked to comment on the overlap between related submissions (http://journals.plos.org/plosone/s/submission-guidelines#loc-related-manuscripts).]

We will carefully review your manuscript upon resubmission and further consideration of the manuscript is dependent on the text overlap being addressed in full. Please ensure that your revision is thorough as failure to address the concerns to our satisfaction may result in your submission not being considered further.

3. We note that the grant information you provided in the ‘Funding Information’ and ‘Financial Disclosure’ sections do not match. 

When you resubmit, please ensure that you provide the correct grant numbers for the awards you received for your study in the ‘Funding Information’ section.

4. We note that you have stated that you will provide repository information for your data at acceptance. Should your manuscript be accepted for publication, we will hold it until you provide the relevant accession numbers or DOIs necessary to access your data. If you wish to make changes to your Data Availability statement, please describe these changes in your cover letter and we will update your Data Availability statement to reflect the information you provide.

5. PLOS requires an ORCID iD for the corresponding author in Editorial Manager on papers submitted after December 6th, 2016. Please ensure that you have an ORCID iD and that it is validated in Editorial Manager. To do this, go to ‘Update my Information’ (in the upper left-hand corner of the main menu), and click on the Fetch/Validate link next to the ORCID field. This will take you to the ORCID site and allow you to create a new iD or authenticate a pre-existing iD in Editorial Manager. Please see the following video for instructions on linking an ORCID iD to your Editorial Manager account: https://www.youtube.com/watch?v=_xcclfuvtxQ

6. Please upload a new copy of Figure 9 as the detail is not clear. Please follow the link for more information: "" ext-link-type="uri" xlink:type="simple">https://blogs.plos.org/plos/2019/06/looking-good-tips-for-creating-your-plos-figures-graphics/"" "" ext-link-type="uri" xlink:type="simple">https://blogs.plos.org/plos/2019/06/looking-good-tips-for-creating-your-plos-figures-graphics/""

[Note: HTML markup is below. Please do not edit.]

Reviewers' comments:

Reviewer's Responses to Questions

**Comments to the Author**

1. Is the manuscript technically sound, and do the data support the conclusions?

The manuscript must describe a technically sound piece of scientific research with data that supports the conclusions. Experiments must have been conducted rigorously, with appropriate controls, replication, and sample sizes. The conclusions must be drawn appropriately based on the data presented. 

Reviewer #1: Partly

Reviewer #2: Yes

**********

2. Has the statistical analysis been performed appropriately and rigorously? 

Reviewer #1: Yes

Reviewer #2: Yes

**********

3. Have the authors made all data underlying the findings in their manuscript fully available?

The PLOS Data policy requires authors to make all data underlying the findings described in their manuscript fully available without restriction, with rare exception (please refer to the Data Availability Statement in the manuscript PDF file). The data should be provided as part of the manuscript or its supporting information, or deposited to a public repository. For example, in addition to summary statistics, the data points behind means, medians and variance measures should be available. If there are restrictions on publicly sharing data—e.g. participant privacy or use of data from a third party—those must be specified.

Reviewer #1: Yes

Reviewer #2: Yes

**********

4. Is the manuscript presented in an intelligible fashion and written in standard English?

PLOS ONE does not copyedit accepted manuscripts, so the language in submitted articles must be clear, correct, and unambiguous. Any typographical or grammatical errors should be corrected at revision, so please note any specific errors here.

Reviewer #1: No

Reviewer #2: Yes

**********

5. Review Comments to the Author

Please use the space provided to explain your answers to the questions above. You may also include additional comments for the author, including concerns about dual publication, research ethics, or publication ethics. (Please upload your review as an attachment if it exceeds 20,000 characters)

Reviewer #1: The manuscript entitled ‘Insight into the Effect of Low Temperature Treatment on Trichome Density and Related Differentially Expressed Genes in Chinese Cabbage’ provide valuable information related to trichome formation in Chinese cabbage.

I have few suggestions for the authors before this research get publication.

1: The English writing is very difficult to understand such as ‘Now, all Raw dates obtained by sequencing have been submitted to NCBI, login number: PRJNA842790.’ which might be ‘Raw data’. Therefore, the manuscript needs native English editing.

2: The references are not match with the citation in MS correctly, such as ‘Chinese cabbage trichomes play an obvious role in increasing leaf thickness to reduce heat of epidermis [26], loss of water [27], and defense against insect pathogen invasion and mechanical injury [28-29], but it also affects the consumption quality of cabbage.’ The references of 26 and 27 the authors listed are not mentioned about Chinese cabbage trichomes at all. Check it please.

Ref.

26. Kenzo T, Yoneda R, Azani MA, Majid NM. Changes in leaf water use after removal of leaf lower surface hairs on Mallotus macrostachyus (Euphorbiaceae) in a tropical secondary forest in Malaysia. Journal of forest research. 2018;13(2): 137-142. doi: https://doi.org/10.1007/s10310-008-0062-z.

27. Hamaoka N, Yasui H, Yamagata Y, Inoue Y, Furuya N, Araki T, et al. A hairy-leaf gene,BLANKET LEAF, of wild Oryza nivara increases photosynthetic water use efficiency in rice.Rice. 2017;10(1): 20. doi: 10.1186/s12284-017-0158-1. Epub 2017 May 12. PMID: 28500411;PMCID: PMC5429320.

3: The authors need describe the materials and methods clearly, such as ‘CT and C are treated at room temperature, while LCT and LC are treated at low temperature.’ What are LCT and LC exactly? Moreover, give the numbers of seedlings in each treatment instead of ‘some of’ and ‘the remaining’.

4: ‘After 10 days of treatment at low and normal temperature, 3 cabbage plants were taken from each treatment and 3 leaves were selected from top to bottom.’ Pay attention to Chinese cabbage and cabbage which are totally different.

5: Table 1 need remove to results part.

6:The authors need rewrite the table and figure legends clearly.

7: the most important data analysis process of the final selected 9 genes for qRT-PCR validation is not mentioned in results part.

8: The abstract and conclusion need reorganize carefully.

Reviewer #2: This MS investigated the different expression genes related with trichome density of Chinese cabbage that treated with low temperature by transcriptome analysis. It is valuable for understand the regulating mechanism of trichome density in Chinese cabbage. Following is my suggestions for this MS:

The authors focused on the DEGs derived from comparison groups of ‘CT vs C, LCT vs LC and CT vs LCT’, while the underlying biological significances should be addressed and explained in this MS.

I did not find the result descriptions of Fig 10., looks like this part was missed in Results part or perhaps this is not a necessary Fig to this MS. Fig 11. should include the statistically significant analysis since these 9 genes are significant DEGs selected from RNA-seq data. The ‘Conclusion’ part actually is the ‘Discussion’, please modify accordingly and add the proper conclusion of this MS. In this MS, the low temperature treatment also affected the Chinese cabbage plant growth (size), how to distinguish the DEGs are truly related with trichome density regulation rather than involving in plant growth (size) or growth speed (slow or quick). Besides, this manuscript needs further editing for language and writing quality.

**********

6. PLOS authors have the option to publish the peer review history of their article (what does this mean?). If published, this will include your full peer review and any attached files.

If you choose “no”, your identity will remain anonymous but your review may still be made public.

**Do you want your identity to be public for this peer review?** For information about this choice, including consent withdrawal, please see our Privacy Policy.

Reviewer #1: No

Reviewer #2: No

**********

[NOTE: If reviewer comments were submitted as an attachment file, they will be attached to this email and accessible via the submission site. Please log into your account, locate the manuscript record, and check for the action link "View Attachments". If this link does not appear, there are no attachment files.]

While revising your submission, please upload your figure files to the Preflight Analysis and Conversion Engine (PACE) digital diagnostic tool, https://pacev2.apexcovantage.com/. PACE helps ensure that figures meet PLOS requirements. To use PACE, you must first register as a user. Registration is free. Then, login and navigate to the UPLOAD tab, where you will find detailed instructions on how to use the tool. If you encounter any issues or have any questions when using PACE, please email PLOS at figures@plos.org. Please note that Supporting Information files do not need this step.

# Author comment: Author response to Decision Letter 0

**DOI:** 10.1371/journal.pone.0274530.r002

23 Aug 2022

Dear Academic Editors and reviewers,

I appreciate the comments on my paper from Academic Editors and reviewers very much. According to your opinions, I have carefully revised my paper again. Please see the revised draft for details. The answers to specific questions are as follows:

Question：During our internal evaluation of the manuscript, we found significant text overlap between your submission and previous work in the [introduction, conclusion, etc.].

 The introduction, conclusion etc are revised further.

Reply to Reviewer 1:

Question 1: The English writing is very difficult to understand such as ‘Now, all Raw dates obtained by sequencing have been submitted to NCBI, login number: PRJNA842790.’ which might be ‘Raw data’. Therefore, the manuscript needs native English editing.

The English language expression and format have been further modified.

Question 2: The references are not match with the citation in MS correctly, such as ‘Chinese cabbage trichomes play an obvious role in increasing leaf thickness to reduce heat of epidermis [26], loss of water [27], and defense against insect pathogen invasion and mechanical injury [28-29], but it also affects the consumption quality of cabbage.’ The references of 26 and 27 the authors listed are not mentioned about Chinese cabbage trichomes at all. Check it please.

The two references cited have been removed.

Question 3: The authors need describe the materials and methods clearly, such as ‘CT and C are treated at room temperature, while LCT and LC are treated at low temperature.’ What are LCT and LC exactly? Moreover, give the numbers of seedlings in each treatment instead of ‘some of’ and ‘the remaining’.

 It has been revised. See the revised manuscript for details.

Question 4: ‘After 10 days of treatment at low and normal temperature, 3 cabbage plants were taken from each treatment and 3 leaves were selected from top to bottom.’ Pay attention to Chinese cabbage and cabbage which are totally different.

It is revised to Chinese cabbage.

Question 5: Table 1 need remove to results part.

The primers were placed in the experimental methods section in most articles. 

Question 6: The authors need rewrite the table and figure legends clearly.

These have been revised, please see the revised manuscript that will be uploaded.

Question 7: The most important data analysis process of the final selected 9 genes for qRT-PCR validation is not mentioned in results part.

qRT-PCR analysis has been added to conclusions and results. Detailed in manuscript 257-259 and 332-346.

Question 8: The abstract and conclusion need reorganize carefully.

The abstract and conclusion have been greatly revised.

Reply to Reviewer 2:

Question 1: The authors focused on the DEGs derived from comparison groups of ‘CT vs C, LCT vs LC and CT vs LCT’, while the underlying biological significances should be addressed and explained in this MS.

It has been revised. See the revised manuscript for details.

Question 2: I did not find the result descriptions of Fig 10., looks like this part was missed in Results part or perhaps this is not a necessary Fig to this MS. 

Fig 10 analysis has been added to conclusions and results. Detailed in manuscript 242-247.

Question 3: Fig 11. should include the statistically significant analysis since these 9 genes are significant DEGs selected from RNA-seq data. The ‘Conclusion’ part actually is the ‘Discussion’, please modify accordingly and add the proper conclusion of this MS.

Fig 11 analysis has been added to conclusions. Detailed in manuscript 257-259 and 332-346.

Question 4: In this MS, the low temperature treatment also affected the Chinese cabbage plant growth (size), how to distinguish the DEGs are truly related with trichome density regulation rather than involving in plant growth (size) or growth speed (slow or quick). Besides, this manuscript needs further editing for language and writing quality.

Two reasons for DEGs selection as follows. The first is the comparison between with and without trichome Chinese cabbage, and between room temperature and low temperature, which does not involve the development stage. Secondly, the correlation between trichome and gene expression was analyzed by WGCNA.

I appreciate the reviewer's comments and accept them. Thank you very much for your review.

Attachment Submitted filename: Response to Reviewers.docx Click here for additional data file.

# Peer review: Decision Letter 1

**DOI:** 10.1371/journal.pone.0274530.r003

**Authors:** Maoteng Li

31 Aug 2022

Insight into the effect of low temperature treatment on trichome density and related differentially expressed genes in Chinese cabbage

PONE-D-22-18944R1

Dear Dr. Cheng,

We’re pleased to inform you that your manuscript has been judged scientifically suitable for publication and will be formally accepted for publication once it meets all outstanding technical requirements.

Within one week, you’ll receive an e-mail detailing the required amendments. When these have been addressed, you’ll receive a formal acceptance letter and your manuscript will be scheduled for publication.

An invoice for payment will follow shortly after the formal acceptance. To ensure an efficient process, please log into Editorial Manager at http://www.editorialmanager.com/pone/, click the 'Update My Information' link at the top of the page, and double check that your user information is up-to-date. If you have any billing related questions, please contact our Author Billing department directly at authorbilling@plos.org.

If your institution or institutions have a press office, please notify them about your upcoming paper to help maximize its impact. If they’ll be preparing press materials, please inform our press team as soon as possible -- no later than 48 hours after receiving the formal acceptance. Your manuscript will remain under strict press embargo until 2 pm Eastern Time on the date of publication. For more information, please contact onepress@plos.org.

Kind regards,

Maoteng Li

Academic Editor

PLOS ONE

Additional Editor Comments (optional):

Reviewers' comments:

Reviewer's Responses to Questions

**Comments to the Author**

1. If the authors have adequately addressed your comments raised in a previous round of review and you feel that this manuscript is now acceptable for publication, you may indicate that here to bypass the “Comments to the Author” section, enter your conflict of interest statement in the “Confidential to Editor” section, and submit your "Accept" recommendation.

Reviewer #1: All comments have been addressed

Reviewer #2: All comments have been addressed

**********

2. Is the manuscript technically sound, and do the data support the conclusions?

The manuscript must describe a technically sound piece of scientific research with data that supports the conclusions. Experiments must have been conducted rigorously, with appropriate controls, replication, and sample sizes. The conclusions must be drawn appropriately based on the data presented. 

Reviewer #1: Yes

Reviewer #2: Yes

**********

3. Has the statistical analysis been performed appropriately and rigorously? 

Reviewer #1: Yes

Reviewer #2: Yes

**********

4. Have the authors made all data underlying the findings in their manuscript fully available?

The PLOS Data policy requires authors to make all data underlying the findings described in their manuscript fully available without restriction, with rare exception (please refer to the Data Availability Statement in the manuscript PDF file). The data should be provided as part of the manuscript or its supporting information, or deposited to a public repository. For example, in addition to summary statistics, the data points behind means, medians and variance measures should be available. If there are restrictions on publicly sharing data—e.g. participant privacy or use of data from a third party—those must be specified.

Reviewer #1: Yes

Reviewer #2: Yes

**********

5. Is the manuscript presented in an intelligible fashion and written in standard English?

PLOS ONE does not copyedit accepted manuscripts, so the language in submitted articles must be clear, correct, and unambiguous. Any typographical or grammatical errors should be corrected at revision, so please note any specific errors here.

Reviewer #1: Yes

Reviewer #2: Yes

**********

6. Review Comments to the Author

Please use the space provided to explain your answers to the questions above. You may also include additional comments for the author, including concerns about dual publication, research ethics, or publication ethics. (Please upload your review as an attachment if it exceeds 20,000 characters)

Reviewer #1: figure legends in Fig 1. A, Three visual fields (front, back, and edge) at room temperature (CT, C)of CT, C at room temperature and low temperature (LCT, LC); As I know, it should be adaxial and abaxial instead of front and back, check it please.

Reviewer #2: Minor issues: Line 39 should be '... on the surfaces of plants ...'; In Table 1, the gene ID should be written with italic format; Conclusion and discussion are very different sections in the main text of a manuscript, Line 260 should be Discussion rather than Conclusion.

**********

7. PLOS authors have the option to publish the peer review history of their article (what does this mean?). If published, this will include your full peer review and any attached files.

If you choose “no”, your identity will remain anonymous but your review may still be made public.

**Do you want your identity to be public for this peer review?** For information about this choice, including consent withdrawal, please see our Privacy Policy.

Reviewer #1: No

Reviewer #2: No

**********

# Editor report: Acceptance letter

**DOI:** 10.1371/journal.pone.0274530.r004

**Authors:** Maoteng Li

6 Sep 2022

PONE-D-22-18944R1 

Insight into the effect of low temperature treatment on trichome density and related differentially expressed genes in Chinese cabbage 

Dear Dr. Cheng:

I'm pleased to inform you that your manuscript has been deemed suitable for publication in PLOS ONE. Congratulations! Your manuscript is now with our production department. 

If your institution or institutions have a press office, please let them know about your upcoming paper now to help maximize its impact. If they'll be preparing press materials, please inform our press team within the next 48 hours. Your manuscript will remain under strict press embargo until 2 pm Eastern Time on the date of publication. For more information please contact onepress@plos.org.

If we can help with anything else, please email us at plosone@plos.org. 

Thank you for submitting your work to PLOS ONE and supporting open access. 

Kind regards, 

PLOS ONE Editorial Office Staff

on behalf of

Dr. Maoteng Li 

Academic Editor

PLOS ONE
